# Burnout and Work Demands Predict Reduced Job Satisfaction in Health Professionals Working In a Surgery Clinic

**DOI:** 10.3889/oamjms.2015.020

**Published:** 2015-02-04

**Authors:** Dragan Mijakoski, Jovanka Karadzinska-Bislimovska, Vera Basarovska, Sasho Stoleski, Jordan Minov

**Affiliations:** *Institute for Occupational Health of Republic of Macedonia - Skopje, WHO Collaborating Center, Ga2len Collaborating Center, Skopje, Republic of Macedonia*

**Keywords:** burnout, work demands, job satisfaction, multiple regression, mediation, health professionals, surgery clinic

## Abstract

**BACKGROUND:**

Burnout syndrome develops in health professionals (HPs) as a result of exposure to chronic emotional and interpersonal workplace stressors. Research demonstrates the links between burnout, work demands, and job satisfaction in hospital HPs.

**AIMS:**

To examine the associations between burnout, work demands and job satisfaction, and to demonstrate the mediation effect of emotional exhaustion on the relationship between work demands and job satisfaction in surgery clinic HPs.

**METHODS:**

Maslach Burnout Inventory was used for assessment of burnout. Work demands and job satisfaction were measured with Hospital Experience Scale and Job Satisfaction Survey, respectively. In order to examine the role of emotional exhaustion, depersonalization, and work demands, controlling for age, hospital tenure, and unit tenure, a hierarchical multiple regression models were tested for each job satisfaction factor.

**RESULTS:**

Job satisfaction was negatively predicted by emotional exhaustion. Certain types of work demands negatively predicted different factors of job satisfaction. Emotional exhaustion was a significant partial mediator of the relationship between work demands and job satisfaction.

**CONCLUSIONS:**

Adequate management of work demands, particularly excessive workload, time pressure, and lack of staff can lead to prevention of burnout and reduced job satisfaction in surgery clinic HPs, and contribute to better quality of patient care.

## Introduction

Hospital health professionals (HPs) face a wide range of hazards on the job, including workplace stress [1]. Burnout syndrome, a particular type of prolonged occupational stress, develops in HPs as a result of exposure to chronic emotional and interpersonal workplace stressors [2-4]. It involves overwhelming exhaustion; feelings of frustration, anger, and cynicism; and a sense of ineffectiveness and failure [3]. The job demands/resources model of stress of Demerouti and Bakker (JD-R model) assumes that the core components of burnout (emotional exhaustion and depersonalization) develop in response to demanding aspects of work (high job demands) leading to overtaxing and exhaustion as well as by lack of resources resulting in withdrawal behavior (depersonalization) and disengagement [4, 5, 6]. The experience of exhaustion is defined as central quality of burnout and it reflects the stress dimension of this syndrome. Yet emotional exhaustion does not describe the relationship of workers with their job. However, exhaustion triggers actions towards emotional and cognitive distancing (depersonalization) of workers from the job and service recipients (i.e. patients in the case of HPs) in order to cope with job demands [4].

Different types of job demands (namely, physical, social, emotional, cognitive, and organizational aspects of the work) characterize hospital settings and their job context. These job demands, especially in the context of reduced job resources (e.g., low salary, job insecurity, low participation in decision making, inadequate supervisor and coworker support), require prolonged physical and/or psychological (cognitive and emotional) efforts and skills in workers and result in certain physiological and/or psychological changes (including overtaxing, exhaustion, cynical attitude towards work, depersonalization, and disengagement) [5-7].

In many studies, job stress and burnout on the organizational level has been associated with various forms of negative responses to the job [8], including low organizational commitment, increased absenteeism [9], lower self-evaluated performances, intention to leave the job [10, 11], turnover, and reduced job satisfaction (or job dissatisfaction) [12-15].

Within organizational context, job satisfaction refers to the extent to which work is a source of need fulfillment (for example, having confidence in leadership; feelings of being valued in the organization; feelings of being part of a team; cooperative spirit between staff members; supervisors that give support and respect workers; adequate ongoing training; fair salary; overall benefits in the organization, etc.) and contentment and this component of the work life keeps people job involving [16]. Both burnout and reduced job satisfaction are clearly linked with high job demands [17]. Conversely, lower job demands have been shown to be associated with higher levels of job satisfaction [18]. However, scientific literature also demonstrated reverse pathways where reduced job satisfaction predicted burnout [19].

Actual study analyzes HPs working in a surgery clinic in Skopje, Macedonia. Health care system in the Republic of Macedonia faces continuous reforms oriented towards improvement of safety, effectiveness, patient-centeredness, timeliness, efficiency, and equity of patient care. These structural changes within health care system together with social reforms aimed to EU accession have resulted in complex hospital reorganisation leading to increased work demands in hospital HPs [20, 21]. The hospital which HPs we analyzed in the actual paper is surgery clinic, an educational base of the Faculty of Medicine in Skopje, providing health care to the general population at secondary and tertiary level.

Very few studies have examined work demands and burnout as predictors of job satisfaction in the region of South-East Europe (SEE). Also, there is a poor pool of data for SEE countries concerning mediation effect of emotional exhaustion on the relationship between work demands and job satisfaction.

The purpose of the present study was to examine:

- the associations between burnout dimensions, work demands, and factors of job satisfaction, taking into account the effects of age, hospital tenure, and unit tenure as potential confounders, and

- the mediation effect of emotional exhaustion on the relationship between predictor (certain types of work demands) and outcome (job satisfaction factors) variables.

## Methods

This cross-sectional study was conducted during June-August 2014 in a surgery clinic that is educational base of the Faculty of Medicine in Skopje. After the official approval from the Hospital Ethics Committee a self-administered questionnaire was distributed to all HPs (doctors and nurses) in the target hospital who were present at the workplace during summer holydays. Each questionnaire was prefaced with an invitation letter and information about the study.

We used Maslach Burnout Inventory (MBI) [22] for the assessment of burnout. Emotional exhaustion (nine items, α=0.9) and depersonalisation (five items, α=0.71) subscales were measured with a 7-point Likert scale (0 = never to 6 every day). The sum score was calculated for each burnout dimension.

Work demands were assessed with the Hospital Experience Scale (HES), a questionnaire constructed through qualitative analysis of focus groups (FGs) for the purposes of FP7 ORCAB Project (http://orcab.web.auth.gr/). The purpose of FGs sessions was to understand what kind of stressors HPs are faced with. Three FGs were planned and conducted in each ORCAB country (one for doctors, one for nurses, and one for residents and interns). The FGs, made up of 6-8 participants, were audio recorded with the permission of the participants and later transcribed. Thematic analysis was used as a main method of qualitative analysis of obtained data. The analysis highlighted the main sources of stress for HPs and thay were used for the development of HES items. The items were categorised into four subscales: physical workload (seven items, α = 0.73), organisational (six items, α = 0.79), emotional (six items, α = 0.75) and cognitive (five items, α = 0.69) work demands. Extensive information on the validation of the HES can be provided upon request. Participants indicated their level of agreement with the items on a 5-point Likert scale (1 = never to 5 = always). The mean score was calculated for each of the four types of work demands. Higher mean score means higher perceived level of particular work demands type.

Job satisfaction was measured with the Job Satisfaction Survey (JSS) [23]. The items of the JSS instrument (39 items) are divided into seven subscales, each representing one of the seven job satisfaction factors: planning issues (five items; ex., confidence in leadership, adequate planning) (α = 0.85), general attitudes (five items; ex., proud to work, good conditions, feel valued) (α = 0.76), performance issues (six items; ex., job is secure, feel part of team, cooperative spirit) (α = 0.84), management issues (five items; ex., given recognition, feel trust, quality priority) (α = 0.85), supervisory issues (six items; ex., gives support, respects, treats fairly) (α = 0.93), training and salary issues (five items; ex., adequate ongoing training, fair salary) (α = 0.87), and benefits issues (seven items; ex., overall benefits, vacation, sick leave) (α = 0.90). Participants were asked to indicate their level of agreement with the items (1 = strongly agree, 2 = agree, 3 = neither agree nor disagree, 4 = disagree, 5 = strongly disagree). Prior to analysis, the answers were re-coded (1=5, 2=4, 3=3, 4=2, 5=1) in order to obtain higher points for higher level of agreement and vice versa because all items are stated in positive direction (ex., there is adequate planning of hospital objectives; I believe my job is secure). Points for statements related to each of the job satisfaction factors were averaged to derive the seven factors of job satisfaction. Higher mean score means higher level of satisfaction with certain JS factor.

The final sample consisted of 160 health professionals (68% females, 32% males) working in a surgery clinic. The response rate was about 80% of HPs who were present at the workplace.

Participants had an average age of 44.85 (SD = 9.6) years and they worked for an average of 199.12 (SD = 138.82) months at the same hospital and 131.48 (SD = 126.47) months within the same unit. About 70% of all participants were married or lived together with their partner and about 90% of them reported full-time contract as a type of employment.

### Analysis

Bivariate analyses were conducted to examine the associations between burnout dimensions, work demands, and job satisfaction factors. Additionally, in order to examine the role of emotional exhaustion, depersonalization, and work demands, controlling for age, hospital tenure, and unit tenure, a hierarchical multiple regression models were tested for each job satisfaction factor. Age, hospital tenure, and unit tenure were entered in the first step, work demands were entered in the second step, while emotional exhaustion and depersonalization were entered in the third step.

Finally, we used the most significant predictors of each job satisfaction factor to fit regression models analysing emotional exhaustion as a mediating variable and its significance in the models. Therefore, a series of regression models were fitted for each job satisfaction factor: model 1 predicting the mediator variable (emotional exhaustion) using the independent variable (certain type of work demands); model 2 predicting the dependent variable (particular job satisfaction factor) using both the independent variable and the mediator; and model 3 predicting dependent variable by using the independent variable.

## Results

The mean score for emotional exhaustion was 18.15 ± 12.8 (range 0-51) and the mean score for depersonalization was 3.51 ± 4.51 (range 0-24). The average scores for physical, organizational, emotional, and cognitive work demands were 3.48 (SD = 0.66), 2.58 (SD = 0.83), 2.32 (SD = 0.72), and 2.88 (SD = 0.79), respectively (on the scale from 1 = never to 5 = always). Participants showed medium satisfaction with all factors representing job satisfaction. The average scores for job satisfaction factors ranged from 3.15 (SD = 0.86) for performance issues to 3.73 (SD = 0.9) for supervisory issues (on the re-coded scale from 1 = strongly disagree to 5 = strongly agree).

Within study sample all job satisfaction factors were negatively correlated with both burnout dimensions. All job satisfaction factors were also negatively correlated with all types of work demands except job satisfaction factor - benefits issues that was negatively correlated with only physical and organizational work demands.

Table 1 shows the standardized beta coefficients for the independent predictors of JS factor planning issues in surgery clinic HPs. Results showed that emotional (*β* = -0.27, *p* < 0.01) and organizational (*β* = -0.22, *p* < 0.05) work demands as well as emotional exhaustion (*β* = -0.26, *p* < 0.05) negatively predicted planning issues (*R*^2^ for the model = 0.350).

**Table 1:** Hierarchical multiple regression model for job satisfaction factor - planning issues in surgery clinic HPs.

Job satisfaction factor - Planning Issues		B	SE	95% CI for B		Beta	*R*^2^
**Step 1**				**Lower**	**Upper**		0.007

	Age	-0.002	0.01	-0.03	0.03	-0.02	

	Hospital Tenure	0.001	0.001	-0.001	0.003	0.12	

	Unit Tenure	0.000	0.001	-0.002	0.001	-0.06	

	Constant	3.60	0.47	2.66	4.54		

**Step 2**	Age	-0.002	0.01	-0.03	0.02	-0.03	0.287

	Hospital Tenure	0.000	0.001	-0.002	0.002	0.04	

	Unit Tenure	-3.79E-5	0.001	-0.002	0.001	-0.01	

	**Organizational Demands**	**-0.26**	**0.12**	**-0.5**	**-0.02**	-0.24*	

	**Emotional Demands**	**-0.39**	**0.13**	**-0.64**	**-0.14**	-0.32**	

	Physical Demands	-0.13	0.13	-0.39	0.13	-0.10	

	Cognitive Demands	0.02	0.12	-0.22	0.27	0.02	

	Constant	5.63	0.57	4.49	6.76		

**Step 3**	Age	-0.002	0.01	-0.03	0.02	-0.02	0.350

	Hospital Tenure	0.001	0.001	-0.001	0.002	0.09	

	Unit Tenure	0.000	0.001	-0.002	0.001	-0.06	

	**Organizational Demands**	**-0.23**	**0.12**	**-0.47**	**-0.002**	-0.22*	

	**Emotional Demands**	**-0.33**	**0.12**	**-0.58**	**-0.08**	-0.27**	

	Physical Demands	-0.03	0.13	-0.29	0.23	-0.02	

	Cognitive Demands	0.07	0.12	-0.16	0.31	0.07	

	**Emotional Exhaustion**	**-0.02**	**0.01**	**-0.03**	**-0.004**	-0.26*	

	Depersonalization	-0.01	0.02	-0.05	0.03	-0.06	

	Constant	5.23	0.57	4.11	6.36		

*R*^2^ = .007 for Step 1; Δ *R*^2^ = .281 for Step 2 (*P*<0.001); Δ *R*^2^ = .062 for Step 3 (*P*<0.01);

* *p*<0.05,

** *p*<0.01.

Table 2 demonstrates that organizational demands (*β* = -0.28, *p* < 0.01) and emotional exhaustion (*β* = -0.44, *p* < 0.001) negatively predicted JS factor general attitudes (*R*^2^ for the model = 0.443).

**Table 2:** Hierarchical multiple regression model for job satisfaction factor - general attitudes in surgery clinic HPs.

Job satisfaction factor - General Attitudes		B	SE	95% CI for B		Beta	*R*^2^
**Step 1**				**Lower**	**Upper**		<0.001

	Age	0.001	0.01	-0.02	0.03	0.01	

	Hospital Tenure	-1.81E-5	0.001	-0.002	0.002	-0.003	

	Unit Tenure	0.000	0.001	-0.002	0.001	-0.02	

	Constant	3.57	0.45	2.68	4.45		

**Step 2**	Age	-0.001	0.01	-0.02	0.02	-0.01	0.285

	Hospital Tenure	0.000	0.001	-0.002	0.001	-0.06	

	Unit Tenure	7.73E-5	0.001	-0.001	0.002	0.01	

	**Organizational Demands**	**-0.32**	**0.11**	**-0.55**	**-0.1**	-0.32*	

	Emotional Demands	-0.21	0.12	-0.45	0.02	-0.19	

	Physical Demands	-0.17	0.12	-0.41	0.07	-0.14	

	Cognitive Demands	-0.01	0.12	-0.24	0.22	-0.01	

	Constant	5.64	0.54	4.58	6.71		

**Step 3**	Age	-5.97E-6	0.01	-0.02	0.02	0.000	0.443

	Hospital Tenure	0.000	0.001	-0.001	0.002	0.03	

	Unit Tenure	-0.001	0.001	-0.002	0.001	-0.08	

	**Organizational Demands**	**-0.28**	**0.10**	**-0.49**	**-0.08**	-0.28*	

	Emotional Demands	-0.12	0.11	-0.34	0.09	-0.11	

	Physical Demands	-0.02	0.11	-0.24	0.21	-0.01	

	Cognitive Demands	0.07	0.10	-0.14	0.27	0.06	

	**Emotional Exhaustion**	**-0.03**	**0.01**	**-0.04**	**-0.02**	-0.44**	

	Depersonalization	-0.01	0.02	-0.04	0.02	-0.06	

	Constant	5.04	0.49	4.07	6.02		

*R*^2^ = .000 for Step 1; Δ *R*^2^ = .285 for Step 2 (*P*<0.001); Δ *R*^2^ = .158 for Step 3 (*P*<0.001);

* *p*<0.01,

** *p*<0.001.

Organizational demands (*β* = -0.23, *p* < 0.05) and emotional exhaustion (*β* = -0.34, *p* < 0.001) were significant predictors of JS factor performance issues (*R*^2^ for the model = 0.418) (Table 3). On the other hand, emotional exhaustion (*β* = -0.40, *p* < 0.001) was the sole predictor of JS factor management issues (*R*^2^ for the model = 0.29) (Table 4). Emotional exhaustion was also the sole negative predictor of JS factors - supervisory issues (*β* = -0.29, *p* < 0.05) (*R*^2^ for the model = 0.22) (Table 5) and training and salary issues (*β* = -0.39, *p* < 0.001) (*R*^2^ for the model = 0.36) (Table 6).

**Table 3:** Hierarchical multiple regression model for job satisfaction factor - performance issues in surgery clinic HPs.

Job satisfaction factor - Performance Issues		B	SE	95% CI for B		Beta	*R*^2^
**Step 1**				**Lower**	**Upper**		0.011

	Age	0.01	0.01	-0.01	0.04	0.14	

	Hospital Tenure	0.000	0.001	-0.002	0.002	-0.03	

	Unit Tenure	0.000	0.001	-0.002	0.001	-0.04	

	Constant	2.67	0.47	1.73	3.60		

**Step 2**	Age	0.01	0.01	-0.01	0.04	0.12	0.284

	Hospital Tenure	-0.001	0.001	-0.002	0.001	-0.09	

	Unit Tenure	-2.78E-5	0.001	-0.002	0.001	-0.004	

	**Organizational Demands**	**-0.30**	**0.12**	**-0.54**	**-0.06**	-0.28*	

	**Emotional Demands**	**-0.26**	**0.13**	**-0.51**	**-0.01**	-0.21*	

	Physical Demands	-0.19	0.13	-0.45	0.07	-0.14	

	Cognitive Demands	-0.02	0.12	-0.26	0.23	-0.01	

	Constant	4.85	0.57	3.71	5.98		

**Step 3**	Age	0.01	0.01	-0.01	0.03	0.12	0.418

	Hospital Tenure	-1.06E-5	0.001	-0.002	0.002	-0.002	

	Unit Tenure	-0.001	0.001	-0.002	0.001	-0.09	

	**Organizational Demands**	**-0.25**	**0.11**	**-0.47**	**-0.03**	-0.23*	

	Emotional Demands	-0.16	0.12	-0.39	0.07	-0.13	

	Physical Demands	-0.06	0.12	-0.3	0.19	-0.04	

	Cognitive Demands	0.06	0.11	-0.17	0.29	0.05	

	**Emotional Exhaustion**	**-0.02**	**0.01**	**-0.04**	**-0.01**	-0.34**	

	Depersonalization	-0.03	0.02	-0.06	0.01	-0.15	

	Constant	4.31	0.54	3.25	5.37		

*R*^2^ = .011 for Step 1; Δ *R*^2^ = .273 for Step 2 (*P*<0.001); Δ *R*^2^ = .134 for Step 3 (*P*<0.001);

* *p*<0.05,

** *p*<0.001.

**Table 4:** Hierarchical multiple regression model for jobsatisfaction factor - management issues in surgery clinic HPs.

Job satisfaction factor - Management Issues		B	SE	95% CI for B		Beta	*R*^2^
**Step 1**				**Lower**	**Upper**		0.002

	Age	0.003	0.01	-0.02	0.03	0.03	

	Hospital Tenure	3.61E-5	0.001	-0.002	0.002	0.01	

	Unit Tenure	0.000	0.001	-0.002	0.002	0.02	

	Constant	3.48	0.46	2.57	4.39		

**Step 2**	Age	0.002	0.01	-0.02	0.03	0.02	0.169

	Hospital Tenure	0.000	0.001	-0.002	0.002	-0.06	

	Unit Tenure	0.000	0.001	-0.001	0.002	0.06	

	Organizational Demands	-0.20	0.13	-0.45	0.05	-0.19	

	**Emotional Demands**	**-0.28**	**0.13**	**-0.54**	**-0.02**	-0.24*	

	Physical Demands	-0.09	0.14	-0.36	0.18	-0.07	

	Cognitive Demands	0.000	0.13	-0.25	0.25	0.000	

	Constant	5.01	0.6	3.82	6.19		

**Step 3**	Age	0.004	0.01	-0.02	0.03	0.04	0.290

	Hospital Tenure	9.41E-5	0.001	-0.002	0.002	0.01	

	Unit Tenure	0.000	0.001	-0.002	0.001	-0.02	

	Organizational Demands	-0.17	0.12	-0.40	0.07	-0.16	

	Emotional Demands	-0.20	0.13	-0.45	0.05	-0.17	

	Physical Demands	0.05	0.13	-0.21	0.31	0.04	

	Cognitive Demands	0.07	0.12	-0.17	0.31	0.06	

	**Emotional Exhaustion**	**-0.03**	**0.01**	**-0.04**	**-0.01**	-0.40**	

	Depersonalization	-0.003	0.02	-0.04	0.03	-0.02	

	Constant	4.46	0.57	3.32	5.59		

*R*^2^ = .002 for Step 1; Δ *R*^2^ = .167 for Step 2 (*P*<0.001); Δ *R*^2^ = .121 for Step 3 (*P*<0.001);

* *p*<0.05,

** *p*<0.001.

**Table 5:** Hierarchical multiple regression model for job satisfaction factor - supervisory issues in surgery clinic HPs.

Job satisfaction factor - Supervisory Issues		B	SE	95% CI for B		Beta	*R*^2^
**Step 1**				**Lower**	**Upper**		0.004

	Age	-0.003	0.02	-0.03	0.03	-0.03	

	Hospital Tenure	0.000	0.001	-0.003	0.002	-0.05	

	Unit Tenure	0.000	0.001	-0.002	0.002	0.04	

	Constant	3.91	0.51	2.91	4.92		

**Step 2**	Age	-0.01	0.01	-0.03	0.02	-0.06	0.143

	Hospital Tenure	-0.001	0.001	-0.003	0.002	-0.08	

	Unit Tenure	0.000	0.001	-0.001	0.002	0.06	

	**Organizational Demands**	**-0.29**	**014**	**-0.57**	**-0.01**	-0.25*	

	Emotional Demands	-0.13	0.15	-0.42	0.17	-0.09	

	Physical Demands	-0.1	0.15	-0.40	0.20	-0.07	

	Cognitive Demands	-0.4	0.14	-0.32	0.25	-0.03	

	Constant	5.53	0.67	4.20	6.86		

**Step 3**	Age	-0.01	0.01	-0.03	0.02	-0.05	0.215

	Hospital Tenure	0.000	0.001	-0.002	0.002	-0.02	

	Unit Tenure	1.66E-5	0.001	-0.002	0.002	0.002	

	Organizational Demands	-0.26	0.14	-0.53	0.02	-0.22	

	Emotional Demands	-0.05	0.15	-0.34	0.24	-0.04	

	Physical Demands	0.02	0.15	-0.29	0.32	0.01	

	Cognitive Demands	0.02	0.14	-0.26	0.3	0.02	

	**Emotional Exhaustion**	**-0.02**	**0.01**	**-0.04**	**-0.01**	-0.29*	

	Depersonalization	-0.01	0.02	-0.06	0.03	-0.06	

	Constant	5.07	0.67	3.75	6.39		

*R*^2^ = .004 for Step 1; Δ *R*^2^ = .139 for Step 2 (*P*<0.01); Δ *R*^2^ = .072 for Step 3 (*P*<0.01);

* *p*<0.05

**Table 6:** Hierarchical multiple regression model for job satisfaction factor - training and salary in surgery clinic HPs.

Job satisfaction factor - Training and Salary		B	SE	95% CI for B		Beta	*R*^2^
**Step 1**				**Lower**	**Upper**		0.016

	Age	-0.01	0.01	-0.03	0.02	-0.05	

	Hospital Tenure	0.001	0.001	-0.001	0.004	0.19	

	Unit Tenure	-0.001	0.001	-0.003	0.001	-0.12	

	Constant	3.34	0.49	2.36	4.32		

**Step 2**	Age	-0.01	0.01	-0.03	0.02	-0.06	0.246

	Hospital Tenure	0.001	0.001	-0.001	0.003	0.13	

	Unit Tenure	-0.001	0.001	-0.002	0.001	-0.09	

	**Organizational Demands**	**-0.23**	**0.13**	**-0.49**	**0.03**	**-0.20**	

	Emotional Demands	-0.28	0.14	-0.55	-0.01	-0.22*	

	Physical Demands	-0.28	0.14	-0.56	-0.01	-0.20*	

	Cognitive Demands	0.04	0.13	-0.22	0.31	0.04	

	Constant	5.54	0.62	4.31	6.76		

**Step 3**	Age	-0.004	0.01	-0.03	0.02	-0.04	0.360

	Hospital Tenure	0.001	0.001	0.000	0.003	0.20	

	Unit Tenure	-0.001	0.001	-0.003	0.000	-0.17	

	Organizational Demands	-0.2	0.12	-0.44	0.04	-0.18	

	Emotional Demands	-0.2	0.13	-0.46	0.06	-0.16	

	Physical Demands	-0.13	0.14	-0.4	0.14	-0.09	

	Cognitive Demands	0.11	0.12	-0.13	0.36	0.10	

	**Emotional Exhaustion**	**-0.03**	**0.01**	**-0.04**	**-0.01**	-0.39**	

	Depersonalization	-0.002	0.02	-0.04	0.04	-0.01	

	Constant	4.95	0.59	3.79	6.12		

*R*^2^ = .016 for Step 1; Δ *R*^2^ = .230 for Step 2 (*P*<0.001); Δ *R*^2^ = .114 for Step 3 (*P*<0.001);

* *p*<0.05,

** *p*<0.001

Both physical work demands (*β* = -0.24, *p* < 0.05) and emotional exhaustion (*β* = -0.31, *p* < 0.01) negatively predicted JS factor benefits issues (*R*^2^ for the model = 0.27) (Table 7).

**Table 7:** Hierarchical multiple regression model for job satisfaction factor - benefits issues in surgery clinic HPs.

Job satisfaction factor - Benefits Issues		B	SE	95% CI for B		Beta	*R*^2^
**Step 1**				**Lower**	**Upper**		0.002

	Age	0.01	0.01	-0.02	0.03	0.06	

	Hospital Tenure	0.000	0.001	-0.002	0.002	-0.03	

	Unit Tenure	4.01E-5	0.001	-0.002	0.002	0.01	

	Constant	3.30	0.47	2.37	4.22		

**Step 2**	Age	0.003	0.01	-0.02	0.03	0.03	0.167

	Hospital Tenure	0.000	0.001	-0.002	0.002	-0.02	

	Unit Tenure	3.19E-5	0.001	-0.002	0.002	0.01	

	Organizational Demands	-0.10	0.11	-0.32	0.12	-0.10	

	**Physical Demands**	**-0.44**	**0.13**	**-0.71**	**-0.18**	-0.34**	

	Constant	5.21	0.59	4.04	6.38		

**Step 3**	Age	0.004	0.01	-0.02	0.03	0.04	0.265

	Hospital Tenure	0.000	0.001	-0.002	0.002	0.04	

	Unit Tenure	0.000	0.001	-0.002	0.001	-0.05	

	Organizational Demands	-0.004	0.11	-0.22	0.21	-0.003	

	**Physical Demands**	**-0.31**	**0.13**	**-0.58**	**-0.05**	-0.24*;	

	**Emotional Exhaustion**	**-0.02**	**0.01**	**-0.03**	**-0.01**	-0.31**	

	Depersonalization	-0.02	0.02	-0.05	0.02	-0.08	

	Constant	4.86	0.57	3.73	5.99		

*R*^2^ = .002 for Step 1; Δ *R*^2^ = .165 for Step 2 (*P*<0.001); Δ *R*^2^ = .098 for Step 3 (*P*<0.01);

* *p*<0.05,

** *p*<0.01

Regression models analysing emotional exhaustion as mediating variable showed that there was a significant indirect effect of emotional demands on the job satisfaction factor ***planning issues*** through emotional exhaustion, *b* = -0.154, *BC*α CI (-0.288, -0.067). This represents medium effect, *k*^2^ = 0.13, 95% *BC*α CI (0.061, 0.227). A Sobel test was conducted and we found partial mediation in the model (*z* = - 3.102, *p* = 0.002). It was detected that emotional exhaustion partially mediated the relationship between emotional demands and planning issues. The study also demonstrated significant indirect effect of organizational demands on the job satisfaction factor ***planning issues*** through emotional exhaustion, *b* = -0.137, *BC*α CI (-0.253, -0.064) and this represents medium effect, *k*^2^ = 0.13, 95% *BC*α CI (0.063, 0.217). A Sobel test demonstrated partial mediation in the model (*z* = -3.120, *p* = 0.002) showing that emotional exhaustion partially mediated the relationship between organizational demands and planning issues.

There was a significant indirect effect of organizational demands on the job satisfaction factor ***general attitudes*** through emotional exhaustion, *b* = -0.175, *BC*α CI (-0.307, -0.087). This represents relatively large effect, *k*^2^ = 0.19, 95% *BC*α CI (0.099, 0.296). A Sobel test was conducted and we found partial mediation in the model (*z* = -3.793, *p* = .0001) demonstrating that emotional exhaustion partially mediated the relationship between organizational demands and general attitudes.

The study demonstrated significant indirect effect of organizational demands on the job satisfaction factor ***performance issues*** through emotional exhaustion, *b* = -0.171, *BC*α CI (-0.301, -0.082). This represents relatively large effect, *k*^2^ = 0.17, 95% *BC*α CI (0.089, 0.267). A Sobel test showed partial mediation in the model (*z* = -3.612, *p* = .0003). It was found that emotional exhaustion partially mediated the relationship between organizational demands and performance. There was a significant indirect effect of emotional demands on the job satisfaction factor ***performance issues*** through emotional exhaustion, *b* = -0.208, *BC*α CI (-0.352, -0.108) representing relatively large effect, *k*^2^ = 0.17, 95% *BC*α CI (0.092, 0.275). A Sobel test was conducted and we found partial mediation in the model (*z* = -3.646, *p* = 0.0003). It was demonstrated that emotional exhaustion partially mediated the relationship between emotional demands and performance.

There was a significant indirect effect of emotional demands on the job satisfaction factor ***management issues*** through emotional exhaustion, *b* = -0.193, *BC*α CI (-0.336, -0.097). This represents a medium effect, *k*^2^ = 0.16, 95% *BC*α CI (0.083, 0.256). A Sobel test found partial mediation in the model (*z* = -3.436, *p* = 0.0006) demonstrating that emotional exhaustion partially mediated the relationship between emotional demands and management issues.

The study demonstrated significant indirect effect of organizational demands on the job satisfaction factor ***supervisory issues*** through emotional exhaustion, *b* = -0.138, *BC*α CI (-0.282, -0.056). This represents a medium effect, *k*^2^ = 0.12, 95% *BC*α CI (0.050, 0.218). A Sobel test showed partial mediation in the model (*z* = -2.928, *p* = 0.003). It was found that emotional exhaustion partially mediated the relationship between organizational demands and supervisory issues.

There was a significant indirect effect of emotional demands on the job satisfaction factor ***training and salary*** through emotional exhaustion, *b* = -0.212, *BC*α CI (-0.364, -0.112) representing a medium effect, *k*^2^ = 0.16, 95% *BC*α CI (0.087, 0.259). A Sobel test found partial mediation in the model (*z* = -3.504, *p* = 0.0005) showing that emotional exhaustion partially mediated the relationship between emotional demands and training and salary issues. There was also significant indirect effect of physical demands on the job satisfaction factor ***training and salary*** through emotional exhaustion, *b* = -0.263, *BC*α CI (-0.436, -0.150). This represents large effect, *k*^2^ = 0.18, 95% *BC*α CI (0.108, 0.289). A Sobel test demonstrated partial mediation in the model (*z* = -3.746, *p* = 0.0002) showing that emotional exhaustion partially mediated the relationship between physical demands and training and salary issues.

Finally, the study showed significant indirect effect of physical demands on the job satisfaction factor ***benefits issues*** through emotional exhaustion, *b* = -0.193, *BC*α CI (-0.325, -0.093). This represents a medium effect, *k*^2^ = 0.14, 95% *BC*α CI (0.064, 0.226) and Sobel test showed partial mediation in the model (*z* = -3.193, *p* = 0.001). It was found that emotional exhaustion partially mediated the relationship between physical demands and benefits issues.

## Discussion

Actual study demonstrated lower average emotional exhaustion (18.2) and depersonalisation (3.5) scores unlike previous studies conducted in hospital HPs [24]. These findings, according to the JD-R Model, could be explained by the effects of work demands leading to increased compensatory efforts in HPs aimed to maintain performance level (higher levels of job engagement) and to reduce associated physiological and psychological costs (lower levels of depersonalisation) [25]. Hospital “protective factors” (e.g., support from superiors, independence in decision making), previously discussed with surgery clinic HPs within focus groups sessions, could also have an important role.

Study participants perceived physical occupational stressors as the most important work demands. They emphasized excessive workload, time pressure, and lack of staff and supplies as particularly demanding aspects of their work life, but receiving feedback on performance and training new staff were also important to them. HPs demonstrated medium level of satisfaction with each of the factors representing job satisfaction. They were mostly content with the superiors’ behaviour that gives support to employees, respects them, and treats them fairly.

JD-R Model assumes that exposure to chronic work demands (physical workload, organisational, emotional, and cognitive) may lead to the depletion of energy (emotional exhaustion) [5], but job resources (in this case support from superiors, independence in decision making, as well as respect and fair treatment by superiors) have motivational potential and lead to low levels of depersonalization.

Similar studies analysing work demands and burnout as predictors of job satisfaction as well as studies examining mediation effect of emotional exhaustion on the relationship between work demands and job satisfaction in the SEE Region are rare. The high response rate gave us an opportunity to obtain comprehensive data concerning burnout in HPs working in this hospital.

This study showed that emotional exhaustion was related to reduce job satisfaction in surgery clinic HPs. The more those HPs felt emotionally exhausted, the more they felt dissatisfied by their job. Organizational work demands negatively predicted planning issues, general attitudes, and performance issues. The more that HPs experienced organizational work demands, the less they were satisfied with the planning in the hospital, hospital working conditions, how the work is being valued, job security, and cooperative spirit. Put it another way, the more that HPs felt that there was a strict hierarchy in the hospital with ambiguous roles and problematic communication with hospital management, the more they were dissatisfied with planning, performance, and general attitudes in the hospital. On the other hand, emotional work demands were related to reduce satisfaction with hospital planning. The more that HPs experienced emotional work demands (e.g., lack of cooperation, lot of competitiveness with the colleagues, emotional involvement in work), the less they have confidence in leadership and the less they were satisfied with the adequacy of planning in the hospital. Finally, physical work demands negatively predicted satisfaction with benefits issues. The more that HPs felt that there was excessive workload at the hospital or unit, time pressure, and lack of staff and supplies, the less they were satisfied with overall benefits at the hospital, as well as with vacation and sick leave issues.

Scientific literature clearly demonstrates the links between work demands, emotional exhaustion, and job satisfaction [12-15, 17, 18] and these relationships underline the JD-R Model [5]. Actual study showed that job satisfaction factors were negatively correlated with both burnout dimensions. Negative correlations between job satisfaction and emotional exhaustion as well as between job satisfaction and depersonalization have also been reported in previous studies [26-28].

While numerous studies examined emotional exhaustion as an outcome variable [2, 4, 29-32], fewer studies analysed emotional exhaustion as a predictor of job satisfaction [33, 34]. However, similarly to our findings, USA study showed that emotional exhaustion negatively predicted job satisfaction [35]. Burnout also predicted reduced job satisfaction among nurse educators in Canada [36].

On the other hand, as it was found in this study, Evans et al. demonstrated that job satisfaction was associated with lower work demands [18]. Work demands were also found to be significant predictors of different work/organization outcomes, such as increased absenteeism and intention to quit job, as well as reduced job engagement [37]. Scientific data showed that these work/organization outcomes are related with each other. Accordingly, previous study emphasized that low job satisfaction can lead to increased intention to quit job and more frequent absenteeism [38].

Furthermore, we have found that emotional exhaustion was a significant partial mediator of the relationship between certain types of work demands and analysed factors of job satisfaction. Higher levels of work demands were associated with higher levels of emotional exhaustion, which, in turn, were associated with reduced job satisfaction. Therefore, work demands were directly and indirectly related to job satisfaction through emotional exhaustion. These results indicate that HPs who perceived higher levels of work demands were more emotionally exhausted and consequently were less satisfied with their job. Maintaining low perceptions of work demands among HPs would most likely result in low emotional exhaustion. Low emotional exhaustion in turn will have positive consequences on job satisfaction.

Finally, the pathways between certain types of work demands and different factors of job satisfaction were not fully mediated by emotional exhaustion: there were also direct effects of work demands on job satisfaction. According to the JD-R Model [5, 39], each type of the work demands represents a stressful situation in which HPs may feel deprived and, thus, may be less satisfied with their job. These work demands in the context of reduced job resources may be particularly detrimental to the HPs job satisfaction. In compliance with aforementioned and similar to our findings, Ren et al. [39] found that job demands were significant predictors of reduced job satisfaction.

Findings of the actual study should be interpreted with caution as cross-sectional design is limited with regard to causality. Also, a “healthy worker effect” may have under-estimated the levels of burnout in surgery clinic health professionals.

As a conclusion, this study shows that:

- emotional exhaustion was related to low job satisfaction in surgery clinic health professionals;

- study participants emphasized physical work demands (excessive workload, time pressure, and lack of staff and supplies) as particularly demanding aspects of their work life;

- certain types of work demands predicted reduced job satisfaction; and

- emotional exhaustion was a significant partial mediator of the relationship between certain types of work demands and analysed factors of job satisfaction.

In contrast with the burnout literature, we found lower average emotional exhaustion and depersonalisation scores, mainly because of the effects of work demands leading to increased compensatory efforts in HPs aimed to maintain performance level and to reduce associated physiological and psychological costs. Hospital “protective factors” (e.g., support from superiors, independence in decision making) could also have an important role.

These findings can be used in the development and implementation of specific organizational interventions in the surgery clinic analysed in the study. The interventions should be guided by the findings that both emotional exhaustion and work demands predicted reduced job satisfaction. Providing adequate job demands-resources interaction and management of all types of work demands, particularly physical work demands (excessive workload, time pressure, and lack of staff and supplies) can lead to prevention of burnout and reduced job satisfaction in surgery clinic health professionals, and contribute positively to better quality of patient care.
